# Lipid and Volatile Profiles of Various Goat Primal Cuts: Aspects of Nutritional Value and Flavor/Taste Attributes

**DOI:** 10.3390/foods13030492

**Published:** 2024-02-03

**Authors:** Nachomkamon Saengsuk, Papungkorn Sangsawad, Pramote Paengkoum, Jaksuma Pongsetkul

**Affiliations:** 1 School of Animal Technology and Innovation, Institute of Agricultural Technology, Suranaree University of Technology, Nakhon Ratchasima 30000, Thailand; pinprae.sae@gmail.com (N.S.); papungkorn@sut.ac.th (P.S.); pramote@sut.ac.th (P.P.); 2 School of Food Industry, King Mongkut’s Institute of Technology Ladkrabang, Bangkok 10520, Thailand

**Keywords:** goat meat, primal cuts, lipid, volatile compounds, flavor

## Abstract

The lipid and volatile profiles of goat primal cuts (shoulder, rib, loin, breast, and leg), as well as their potential impact on nutritional and flavor/taste attributes, were investigated. The breast cuts had the lowest protein but the highest fat content. Triacylglycerol was the predominant lipid in all cuts (82.22–88.01%), while the breast cuts had the lowest triacylglycerol and the highest diacylglycerol and free fatty acids. Also, the highest unsaturated fatty acid (UFA), both monounsaturated fatty acid (MUFA) and polyunsaturated fatty acid (PUFA), was obtained in the breast cuts. These findings correlated well with the highest peroxide value (PV) and thiobarbituric acid reactive substances (TBARS) value. The volatile profiles of the various grilled cuts indicated that the breast and leg cuts had similar volatiles, with higher amounts of alcohol, aldehyde, ketone, and ester than others, which could explain the flavor oxidation by lipid and off-flavors in spoiled meat. While the shoulder, rib, and loin cuts had higher amounts of nitrogen-containing compounds. The highest sulfur-containing and hydrocarbon compounds were also observed in the shoulder cuts, which are mainly formed during the Maillard reaction and responsible for the cooked meat flavor. This investigation revealed that each cut of goat meat has a varied composition, especially in lipids and volatile compounds. Thus, meat quality differs in terms of nutritional aspects and flavor/taste characteristics, enabling consumers to select nutritious or proper cuts for their cooking to achieve the most satisfaction from goat meat consumption.

## 1. Introduction

Goat meat is an important source of nutrients as it is a common type of red meat consumed worldwide annually, especially in Southeast Asia, the Caribbean, North Africa, and Middle Eastern countries. Over the last decade, world goat meat production has increased by 41.66%, in which Asia has the largest contribution in total meat production (70.7%) [1]. Compared to other red meats such as pork, beef, and lamb, goat meat or chevon has lower levels of total fat, saturated fat, and cholesterol [1]. These lipid profiles make it an attractive alternative for consumers and a healthier meat compared to other red meats, since it is positively correlated with a reduction in the risk of developing heart diseases, such as coronary artery disease and atherosclerosis [2]. In general, there are many factors that govern the lipid profiles of goat meat. These include age, sex, weight, feed, parts/types of cutting, storage conditions, processing, etc. [3,4].

Not only for the nutritional aspects, but lipid profiles also play a profound role in terms of meat quality. Focus on red muscles, intramuscular fat (IMF), the fat located between and within muscle fibers (cells), has been noted as a critical contributor to eating quality characteristics through the development of tenderness, juiciness, and flavor [2]. The decrease in the shear force value of cooked sheep meat as the IMF content increased resulted in the development of meat tenderness [5]. Also, meat juiciness assessed via sensory evaluation with consumers decreases with the reduction in the IMF content in goat and sheep meat [6]. This reveals the positive correlation between the development of a desirable texture and the IMF contents of the meat. In addition, in the aspects of flavor and taste characteristics, the strong correlation between flavor, which represents the most sensory stimulation changes, and IMF content through volatile compound variation has been noted [3]. In general, PUFAs, i.e., linoleic (C18:2), linolenic (C18:3), arachidonic (C20:4), and eicosapentaenoic acid (C20:5), were obtained as the most prevalent in the IMF of red meats, and it is well known that the degradation of them, particularly via lipid oxidation, occurs easily and results in residual lipid-derived volatiles, such as aldehydes, ketones, alcohols, furans, etc. [7]. Previous studies noted that these volatiles, in both fresh and cooked meat, are strongly responsible for either a desirable or undesirable odor/flavor as well as taste characteristics.

The IMF content in goat meat is approximately 2–5%, which is influenced by the breed, gender, age, feeding, and muscle composition [8]. In fact, goat carcasses can be divided into several cuts (also known as primal cuts or subprimals) for utilization. The rib, located on the animal’s side, is known for its marbling, which adds flavor and tenderness to the meat. The shoulder, obtained from the upper section of the animal’s shoulder, is known as a lean cut, but it typically has tougher meat compared to other cuts. The loin is the region of the body between the ribs and the hindquarters and is also known as another lean cut. The leg is the meat from the hindquarters next to the loin, while the meat from the chest part of a goat is referred to as the breast [8]. Some previous studies reveal the differences in lipid contents and compositions of each primal goat cut. The various four Korean native black goat meat cuts, including loin, hind leg, neck, and foreleg, had different fat compositions [9]. Saturated fatty acids (SFAs) and UFAs were the highest in the rib and hind leg, respectively, when compared to all cuts (*p* < 0.05). Additionally, significant differences in the total fat content and fatty acid composition between fillet and shank goat cuts have been pronounced [10]. The total fats averaged 1.68% in the fillet and 4.02% in the shank, with the total SFAs and PUFA ranging from 42.75–45.23% and 12.35–14.04% in the fillet and shank cuts, respectively. In the present study, five commercial primal cuts including the shoulder, rib, loin, breast, and leg of Thai-native (TN) × Anglo-Nubian (AN) goat were selected. This crossbred goat (TNAN; 50:50) is an important commercial goat breed in Thailand and the most consumed in Southeast Asia. It exhibited more multiple birth rate, a larger body size, and a higher carcass yield. The moisture, protein, fat, and ash content of this crossbred goat ranged from 73–79%, 16–23%, 2–5%, and 1–1.5%, respectively, depending on sex and diet [11]. However, there is a deficiency of information on the chemical composition, particularly the lipid and volatile profiles of the individual cuts that are most commonly consumed and commercially available. Therefore, the information related to the lipid profiles of each primal cut of this goat meat breed is interesting. Moreover, to date, no information on the volatile profiles of each primal cut has been reported. To achieve the maximum value of choosing each goat cut to consume, in the aspects of nutritional value and flavor/taste attributes, therefore, the objective of this present study was to investigate the lipid profiles in relation to the lipid oxidation and volatile compounds of various primal goat cuts.

## 2. Materials and Methods

### 2.1. Sample Collection and Preparation

**Figure 1:**
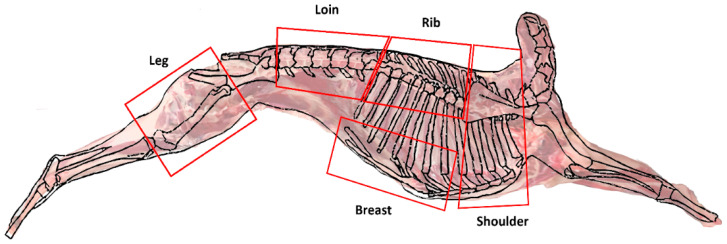
Goat meat primal cuts.

Twenty-five TNAN crossbred goat kids were brought from 3 different farms in the northeast part of Thailand to the SUT farm. They were then randomly allotted into 3 lots/houses, each containing 6–7 animals. Each house (represented as lots of samples) was an enclosed system with adequate ventilation and a slatted floor, allowing adjacent animals to see and interact with each other. The goats were reared indoors on a maize silage-based ration with free access to hay and straw for 4 months until they were ready for slaughter. For primal cut preparation, all 25 goats averaging 17.80 ± 5.43 kg were slaughtered in a commercial slaughterhouse. The carcass, after chilling at 2 °C for 24 h, was dressed into 5 primal cuts including (1) shoulder, (2) rib, (3) loin, (4) breast, and (5) leg, as depicted in Figure 1. All analyses were performed by randomly selecting the cuts from the 3 different lots, and each lot was analyzed with 3 replicates.

Each primal cut was evaluated on its proximate compositions including moisture, protein, fat, and ash content according to AOAC methods [12] with the analytical No. of 35.1.13, 35.1.14, 35.1.25, and 35.1.15, respectively. Cholesterol content was also measured [2]. In brief, the gas chromatograph fitted with a flame ionization detector equipped with an HP-5 column (30 m × 0.32 mm; film thickness, 0.22 µm; Agilent Technologies, Palo Alto, CA, USA) was used for the analysis. The injection port and detector temperature were set at 260 °C and 255 °C, respectively. Cholesterol was identified by comparing the relative retention time of the sample with the α-cholesterol standard (Cargo Erba Reagents, Milan, Italy), calculated, and expressed as mg/100 g meat. Then, the determination of lipid and volatile profiles was further conducted. For the determination of lipid profiles, lipids from each primal cut were extracted using a chloroform–methanol solvent extraction system [13]. Briefly, the goat meat (25 g) was homogenized with 200 mL of a chloroform/methanol/distilled water mixture (50:100:50, *v*/*v*/*v*) at 9500 rpm for 2 min (4 °C). The 50 mL of chloroform was added and homogenized at 9500 rpm for 1 min, followed by adding 25 mL of distilled water and homogenized again for 30 s. The homogenate was then centrifuged at 3000× *g* for 15 min (4 °C) using a refrigerated centrifuge (Beckman Coulter, Avanti J-E Centrifuge, Palo Alto, CA, USA) to obtain the supernatant, which was transferred into a separating flask. The chloroform layer was poured into a 125 mL Erlenmeyer flask with approximately 2–5 g of anhydrous Na_2_SO_4_. After vigorous shaking, the mixture was filtered using a Whatman No. 4 filter paper and transferred into a round-bottom flask. Then, the solvent was evaporated at 25 °C, flushed with nitrogen, sealed tightly, and kept at −40 °C until analyses. For the determination of volatile profiles, the goat meat (150–200 g, 25–30 mm thickness) was grilled on an electric two-plate griddle (900XP, Electrolux, Bangkok, Thailand) at 180 °C until the core temperature reached 80 °C for 3 min (total cooking time was approximately 7–12 min) before analysis [2].

### 2.2. Measurement of Lipid Classes

A thin-layer chromatography/flame ionization detection analyzer (IATROSCAN^®^ TLC/FID Analyzer, IATRON Laboratories, Inc., Tokyo, Japan) was used to determine lipid classes. Lipids (1 µL) were spotted onto the scanned quartz rod (silica powder coated Chrmatorod-S III, IATRON laboratories, Inc., Tokyo, Japan) and separated using a mixture of ether/chloroform/acetic acid (50:20:0.7, *v*/*v*/*v*) for 30 min. Then, samples were dried at 105 °C for 5 min and immediately scanned with the TLC-FID analyzer with a scanning speed of 30 s/scan. The analytical conditions were H_2_, flow rate of 160 mL/min; air, flow rate of 2000 mL/min. The chromatographic peaks of the samples were identified using the retention durations of lipid standards. Based on the peak area ratio, the amount of each lipid was determined and expressed as a percentage of all lipids [13].

### 2.3. Measurement of Fatty Acid Compositions

Prior to analysis, fatty acid methyl esters (FAMEs) were prepared by methylation. The prepared methyl ester was analyzed using gas chromatography (Hewlett-Packard 7890A; Agilent Technologies, Santa Clara, CA, USA) equipped with the flame ionization detector (FID) at a split ratio of 1:20. A fused silica capillary column (SP 2560, Supelco Inc., Bellefonte, PA, USA, 100 m × 0.25 mm i.d., 0.20-µm film thickness) was used. The analytical conditions were an injection port temperature of 250 °C and detector temperature of 270 °C. The oven was programmed from 170 to 225 °C at a rate of 10 °C/min. Retention times of FAME standards (Supelco 37-component FAME mix, including conjugated linoleic acids, from Sigma-Aldrich, Milan, Italy) were used to identify chromatographic peaks of the samples. The fatty acid compositions were reported as g fatty acid/100 g lipid [13].

### 2.4. Measurement of Lipid Oxidation Products (CD, PV, TBARS)

Conjugated diene (CD) was measured [14]. Lipid (0.1 g) was dissolved in 5.0 mL of isooctane and subjected to absorbance measurement at 234 nm using a UV-1601 spectrophotometer (Shimadzu, Japan). Peroxide value (PV) and thiobarbituric acid reactive substances (TBARS) value were measured [15]. For PV measurement, cumene hydroperoxide (0.5–2 ppm) was used for a standard curve (y = 0.5603X + 0.1724, R^2^ = 0.9858) and the results were expressed as mg hydroperoxide/kg sample. For TBARS value, a standard curve of malonaldehyde bis (dimethyl acetal) at 0–2 ppm was prepared (y = 0.1874X + 0.1224, R^2^ = 0.9874) and the results were reported as mg malonaldehyde (MDA)/kg sample.

### 2.5. Measurement of Volatile Profiles (GC-MS)

The volatile compounds of the grilled primal goat meat cuts were determined using a solid-phase microextraction gas chromatography–mass spectrometry (SPME GC-MS) [2]. The minced meat (5 g) was placed in a 20 mL headspace vial. The vials were tightly capped with a PTFE septum and heated at 60 °C with an equilibrium time of 2 h. Then, volatiles were allowed to absorb into an SPME fiber (50/30 lm DVB/Carboxen™/PDMS StableFlex™) (Supelco, Bellefonte, PA, USA) at 60 °C for 1 h. After that, the SPME device was removed, and the samples were immediately inserted into the injection port of the GC-MS. An HP 5890 series II gas chromatography (GC) coupled with HP 5972 mass-selective detector equipped with a splitless injector and coupled with a quadrupole mass detector (Hewlett Packard, Atlanta, GA, USA) was used to separate and identify the volatiles. The compounds were identified using ChemStation Library Search (Wiley 275.L). Quantitative determination was evaluated using an internal calibration curve of the stock solutions of the compounds in ultra-pure water saturated in salt and analyzed by the optimized HS-SPME method, in which the quantification limits were calculated to a signal-to-noise (S/N) ratio of 10. The identified volatile compounds were presented as a percentage of the relative peak area of the total peak.

### 2.6. Statistical Analysis

All analyses were performed in triplicate (*n* = 3) in three lots of samples and reported as means ± SD. Statistical analysis was performed using one-way analysis of variance (ANOVA). Mean comparison was evaluated using Tukey’s test at the significance level of 95% (*p* < 0.05) by the SPSS statistic program (Ver. 20.0) (SPSS Inc., Chicago, IL, USA).

## 3. Results and Discussion

### 3.1. Proximate Composition

Table 1 summarizes the proximate compositions of all five primal goat cuts. It was found that the moisture, protein, and fat content of the shoulder, rib, loin, and leg cuts did not differ significantly (*p* > 0.05). While the breast cuts had the lowest moisture and protein contents, but the highest fat content, compared to the other cuts (*p* < 0.05). Based on a dry basis, all cuts had protein as the major component (16.26–19.05%), indicating that goat meat is generally a good source of protein. The fat content of all cuts was obtained in the range of 1.74–4.35%. This was in the same range as the previous study [11], which investigated the same breed and age as in our study. In general, an inverse relationship between the fat content and the amount of water present in the muscles was noted, which was in good accordance with our results. In fact, all the primal goat cuts can be categorized as lean meat since the IMF contents were lower than 5% [16]. However, there was no significant difference in the ash content among all cuts (*p* > 0.05). The different proximate compositions of the various goat cuts were reported by many previous studies. For instance, the rib cuts of Korean native black goat meat had the lowest moisture and ash contents compared with the loin, hind leg, neck, and foreleg [9]. Subsequently, the highest fat content was found in the rib and a significant variation was also observed among all cuts for the protein content. The hind limb cuts of Bore goat are associated with high value because of the low fat and high protein content of the carcass, compared to the fore limb, neck, ventral trunk, and dorsal trunk [17]. In general, the total fat content of meat muscle affects the fatty acid profiles, thus resulting in different meat characteristics related to lipid-derived products.

The cholesterol content of all goat cuts was found in the range of 62.42–69.02 mg/100 g, as shown in Table 1. Among all cuts, the highest cholesterol content was obtained in the leg cuts (*p* < 0.05). Generally, the cholesterol content of goat meat varies between 30 and 60 mg/100 g, in which the highest cholesterol levels were obtained in leg cuts [18]. This may be due to the higher oxidative muscles in this cut. This explanation coincided with the fact that leg muscles are composed of 76–79% oxidative fibers, whereas breast muscles are composed of glycolytic fibers [19]. The oxidative muscles contain more phospholipids (and in cellular membranes) than the glycolytic muscles, and cholesterol is more likely to bind to these lipids in the cell membranes. Thus, the higher cholesterol levels in the leg cuts are associated with this membrane in the oxidative muscles. However, the results are interesting from the perspective of nutrition because the contents were well below the maximum daily limits for cholesterol intake (300 mg per day) [20]. A portion of 200 g of any goat cuts under consideration refers to less than half of these recommended levels. Moreover, recent research emphasizes that the amount of dietary cholesterol cannot be conclusively linked to consumers’ health. There is ample evidence that SFAs and *trans* fats adversely affect consumers’ health, increasing the risk of cardiovascular diseases. The association between dietary cholesterol and atherogenic effects may be influenced by the fact that foods high in SFAs commonly contain dietary cholesterol. [21].

### 3.2. Lipid Classes

Lipids found in biological systems are oxidizable to varying degrees and consist of one or more of the following classes: mixture of mono-, di-, and triacylglycerols, phospholipids, and free fatty acids [22]. According to Table 1, all the goat cuts contained triacylglycerols as the main component, accounting for 82.22–88.01%. In fact, triacylglycerols constitute approximately 80–95% of the lipid reserves of meat and the amount of IMF is directly correlated with their number [23]. An increase in IMF leads to an increase in the amount of fat cells (triglycerides), but the amount of phospholipids remains the same because the number of membranes is constant [24]. In this study, there were no significant differences in the phospholipid content among all samples (*p* > 0.05), which was found to be in the range of 9.55–10.01% of the total lipids. Our results were slightly different from previous reports since the breast cut, which contained the highest fat content, had a lower triacylglycerol content, compared to the others (*p* < 0.05). However, the results may be coincidental with the higher diacylglycerol and free fatty acid contents of the breast cuts (*p* < 0.05). Moreover, the amount of monoacylglycerol was obtained in the breast cut only. In general, triacylglycerol can hydrolyze by lipases, either endogenous or microbial lipases, and constructs mono- and diacylglycerol, as well as free fatty acids. The hydrolysis of triglycerides and release of fatty acids indicate lipid degradation occurred in the product, which may further contribute to meat nutrition and characteristics. The synthesis of glycidyl esters, which in turn produce 3-MCPD esters (3-monochloropropane-1,2-diol), which are unfavorable in fat, is facilitated by mono- and diacylglycerol. As reported in previous works, of all lipid forms, free fatty acids are the most susceptible to lipid oxidation, followed by phospholipids, compared with the major triacylglycerol [25]. The results indicated that each cut had varying lipid hydrolysis levels, which may be further prone to varying lipid oxidation levels. In general, a moderate amount of lipid oxidation may serve to generate the desirable characteristic flavor of meat and meat products [24]. On the other hand, an excess level may conversely affect undesirable meat characteristics, for example, rancidity, off-flavors, undesirable taste, etc. Therefore, according to our results, breast cuts may experience greater lipid oxidation than other cuts due to their higher contents of mono- and diacylglycerol and free fatty acids when applying the breast cuts for cooking or storage, compared with other cuts.

### 3.3. Fatty Acid Profiles

**Figure 2:**
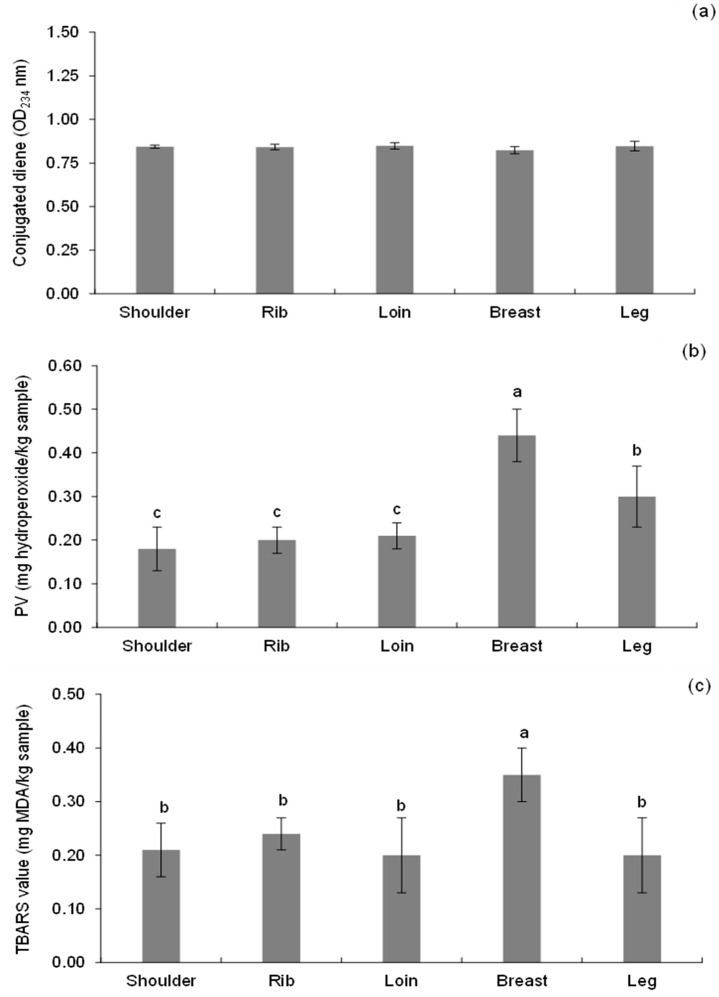
Lipid oxidation products. (**a**) Conjugated diene (OD_234_), (**b**) peroxide value (PV), and (**c**) thiobarbituric acid reactive substances (TBARS) value (**c**) of various goat primal cuts. Different lowercase superscripts indicate the significant difference (*p* < 0.05).

The fatty acid profiles of various primal goat cuts are shown in Table 2. Interestingly, the fatty acid compositions of the shoulder, rib, and loin cuts were similar, with the total SFA accounting for 51.19–52.47 g/100 g lipids as the major compositions. Meanwhile, the fatty acid compositions of the breast and leg cuts were similar, which contained the total MUFA as dominant (53.99–56.85 g/100 g lipids), followed by SFA of 37.31–38.79 g/100 g lipids. However, the total PUFA did not differ between the primal goat cuts (*p* > 0.05). The fatty acid composition of meat is a major determinant of product shelf-life/storage stability and flavor [26]. The results corresponded well with the higher PV and TBARS values of the breast and leg cuts, compared with the others (Figure 2). This was attributable to the fact that the high total UFA of the breast and leg cuts (60.25 and 62.17 g fatty acid/100 g lipid, respectively) accelerated lipid oxidation to the meat higher than other cuts. The previous report stated that the amount of UFA correlates exponentially with lipid oxidation susceptibility. The dissociation energy of carbon–hydrogen bonds is lower in allylic (R1-CH=CH-CH2-R2) and bis-allylic methylene (R1-CH=CH-CH2-CH=CH-R2) positions than in fatty acids without double bonds [27]. As a result, the greater the number of double bonds, the greater the oxidative susceptibility due to the increased number of reaction sites. Previous studies of goat cuts revealed that different cuts had different fatty acid compositions. For instance, four cuts of Korean black goat meat, including loin, hind leg, neck, and foreleg, were found to have different fat compositions [9]. Compared with all cuts, SFA and UFA were highest in the rib and hind leg, respectively (*p* < 0.05). Differences in the total fat content and fatty acid composition between fillet and shank goat cuts have been noted [10]. The fillet and shank cuts contained an average of 1.68 and 4.02% fat, respectively, with a total SFA and PUFA content ranging from 42.75 to 45.23% and 12.35 to 14.04% for the fillet and shank cuts, respectively.

Among all fatty acids, all cuts had oleic acid (C18:1n9), palmitic acid (C16:0), and stearic acid (C18:0) as major fatty acids. The results were correlated with some previous research that reported that these three fatty acids were the major fatty acids found in fillet and shank goat cuts [10], as well as loin, hind leg, neck, or either foreleg [9]. From the nutrition aspect, the PUFA/SFA ratio can be used as an index for determining if the nutritive quality should be high. From this study, all goat cuts had a PUFA/SFA ratio of 0.08–0.15. In general, the PUFA/SFA ratios of goat meat are often higher than similar values for lamb/mutton, beef, and pork [28]. Moreover, a high proportion of n-3 fatty acids within PUFA is beneficial for improving hyperlipidemia, but a low n-6/n-3 ratio (<5) is associated with a decreased risk of cancer [29]. It was found that the shoulder, rib, and loin cuts exhibited higher n-3 proportions than the breast and leg cuts. Also, the formers contained lower n-6/n-3 ratios than the others. As a result of these findings, it is possible to conclude that shoulder, rib, and loin may be the better choice to consume in terms of consumer health.

Furthermore, varying fatty acid compositions of each cut were further responsible for meat characteristics, particularly odor, flavor, or taste since fatty acids are related to the generation of volatile and lipid oxidation products during cooking, as well as their interaction with Maillard reaction products to generate other volatiles that contribute to flavor [2]. Lipid oxidation products that engage in Maillard reactions are very certainly aldehydes that compete for amino compounds with carbonyls from reducing sugars. This rivalry results in distinct and desirable flavor components in cooked meat. It has been noted that UFA is the most important in the flavor formation of meat products [7]. Therefore, the results of the fatty acid compositions were considered in the following sections in terms of their relationship with the lipid oxidation and volatile compounds of cooked meat.

### 3.4. Lipid Oxidation Products

The principal mechanism responsible for the qualitative deterioration of meat and meat products through reducing shelf-life is lipid oxidation [30]. It has an impact on color, texture, nutritional value, taste, and flavor, resulting in rancidity. It is also responsible for off-flavors and unsatisfactory taste, both of which are key reasons for customer rejection. In this study, CD and PV were used as tools to evaluate the primary products, whereas monitoring of the TBARS value represented the secondary product of lipid oxidation.

The CD value was used to determine the abnormal production of conjugated compounds, which is the first sign of the initiation stage of lipid oxidation. During the initiation step, hydrogen is extracted from a UFA. A double-bound rearrangement tends to stabilize the resulting alkyl radical, forming conjugated dienes [31]. The CD values of all primal cuts did not differ significantly (*p* > 0.05), with values ranging from 0.82 to 0.85 OD234 nm (Figure 2a). The existence of CD in all goat cuts indicated that lipid oxidation occurred, probably due to the high content of UFA in the samples and prooxidants in the muscle of red meats. Not only UFA, but also myoglobin and other heme molecules from red meat can serve as prooxidants [32]. However, no difference among all samples may presume that the formation and decomposition of CD took place at equal rates.

Lipid hydroperoxides are the primary products of oxidation, also known as the peroxide value. Figure 2b shows that the breast cuts had the highest PV values (0.44 mg hydroperoxide/kg sample), followed by the leg cuts (0.30 mg hydroperoxide/kg sample), and there was no significant difference in the PV values (0.18–0.21 mg hydroperoxide/kg sample) between the shoulder, rib, and loin cuts (*p* > 0.05). The amount of UFA has a positive correlation with the PV values; therefore, these findings corresponded with the fatty acid composition (Table 2), which revealed that the goat breast and leg cuts had the highest total UFA compared to the other goat cuts. The results may presume that lipid oxidation/degradation in breast and leg cuts can occur at a faster rate, compared to shoulder, rib, or loin cuts, which may further respond to the different deterioration rates of the meat, in terms of rancidity or undesirable odor/flavor. However, CD and PV may not adequately indicate the quantity of lipid oxidation since hydroperoxides are rapidly broken down into secondary compounds. In fact, the formation of CD occurs at the early stages of lipid oxidation and hydroperoxides are expected to decompose to secondary products.

The decrease or reaching a stagnant level in CD and PV were typically accompanied by an increase in TBARS. Malondialdehyde or MDA (1,3-propanedial) is one of the most important aldehydes formed during the secondary lipid oxidation of PUFA. This aldehyde is also important in meat since it causes rancid aromas at low concentrations and has been shown to be the main indicator of lipid oxidation [23]. Figure 2c depicts the TBARS value of several goat primal cuts. The breast cuts had the highest TBARS value (0.35 mg MDA/kg sample) (*p* < 0.05), while there was no difference in the TBARS value among the shoulder, rib, loin, and leg cuts (*p* > 0.05). The results indicated that lipid oxidation occurred at the highest level in the breast cuts, compared to the others, corresponding well with the highest UFA found in this sample. However, various studies identified values of 2–2.25 mg MDA/kg per sample as the recognized limit for no rancidity in meat and meat products [2,4,15]. As a consequence, the TBARS value in all the goat primal cuts ranged from 0.20 to 0.35 mg MDA/kg per sample, which did not exceed the approved limit value. Although the TBARS value of the goat primal cuts was within the limit, the initial TBARS value of meat has significance because it can rapidly increase during cooking and storage.

### 3.5. Volatile Compounds

**Figure 3:**
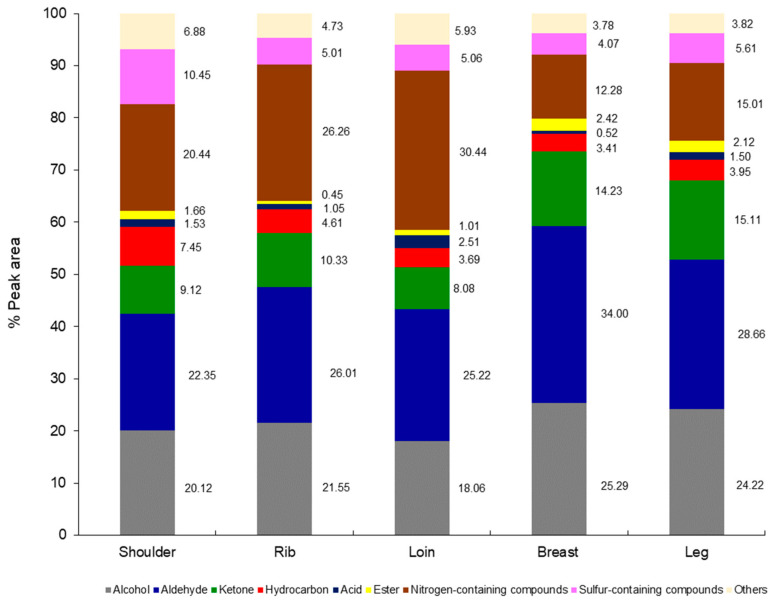
Total intensity of volatiles (% peak area) derived from various grilled goat primal cuts. Alcohol, aldehyde, ketone, hydrocarbon, acid, and ester referred to lipid-derived compounds.

Various goat primal cuts were grilled and subjected to determine their volatile compounds using SPME-GCMS. Based on their chemical functions, the compounds were classified into nine groups: alcohol, aldehyde, ketone, hydrocarbon, acid, ester, nitrogen- and sulfur-containing compounds, and others, as shown in Figure 3. It was found that all cuts contained alcohols and aldehydes as major volatiles, accounting for more than 20% of the total peak area. However, each cut contained various proportions of each volatile group, i.e., the breast and leg cuts had a higher proportion of aldehydes and ketones than the shoulder, rib, and loin cuts. While all three of the latter contained a higher proportion of nitrogen-containing compounds than the breast and leg cuts. The results greatly correlated with the difference in their chemical compositions, particularly the protein and fat contents, described as follows.

In general, organic compounds in meat, such as amino acids, reducing sugars, and fatty acids, undergo chemical changes during cooking that are accelerated by heat, resulting in non-volatile and volatile flavor compounds. For volatile compounds, it was noted that they were mainly produced by the thermal oxidation of lipids [7]. This was consistent with our results; among all volatiles, alcohol, aldehyde, ketone, hydrocarbon, acid, and ester are referred to as “lipid-derived compounds”. It can be presumed that lipid compositions play a profound role in the odor/flavor of grilled goat meat since it contained more than 60% of the total volatiles.

Each primal cut had a different fat content and composition, thus resulting in different lipid-derived volatile profiles. With multiple unsaturated bonds, PUFA is susceptible to oxidation, resulting in a complex variety of volatile secondary oxidation products that generate particularly unpleasant off-flavors in meat products. Although saturated fatty acids are considered to be more stable than UFAs, when heated to 150 °C or higher, such as on the surface of grilled steaks, SFA oxidizes in a more complex pattern, generating long-chain alkanes, alkanals, and ketones [33]. Carbonyl compounds, such as aldehydes and ketones, are mostly responsible for the oxidized flavor caused by lipid oxidation; however, hydrocarbons (alkanes, alkenes, and alkylfurans) and alcohols play a smaller role [34]. As shown in Table 3, the grilled goat cuts included twelve aldehydes, the majority of which were produced from the oxidation of C18 and C20 UFAs [35]. Among the aldehyde groups, hexanal was the most prevalent aldehyde in all the grilled goat cuts (>10% of total peak area), followed by pentanal and nonanal. The oxidation of C18 UFA produces aldehyde compounds such as hexanal, heptanal, octanal, nonanal, 2-octenal, and 2-decanal [34]. This corresponded well with the lipid compositions, which depicted that all goat cuts had the compositions of C18 fatty acids, both SFA and UFA, as high amounts (Table 2). The previous study reported that pentanal, hexanal, heptanal, and nonanal were detected as warmed-over flavors (WOFs) in grilled goat meat, and hexanal was found to be the main contributor among all compounds [36]. WOF is an indicator used to detect an unpleasant flavor characteristic usually associated with cooked meat [37]. Among all cuts, the breast cuts had the greatest total aldehydes, followed by the leg, rib, loin, and shoulder cuts, respectively. Also, the breast cuts gave more 3-methyl-butanal than the others (6.02% of total peak area). 3-Methyl-butanal is a strong flavor compound that occurs in the Maillard reaction and is responsible for the meaty note in toasted or grilled flavors [38]. Moreover, the 2-octenal was only observed in the breast cuts, associated with the highest concentration of linoleic (n-6) of this sample (Table 2). Varlet and Fernandez [39] stated that 2-octenal is a product of oxidation of n-6 PUFA, such as arachidonic acid, which gives green aromatic notes. In general, aldehydes are the most prominent volatiles produced during lipid oxidation; therefore, the greatest amount of aldehydes in the breast cuts correlated well with the highest PV and TBARS values of this sample (Figure 2b,c).

The total ketones had a similar trend to the total aldehydes; the grilled breast and leg cuts of goat had greater total ketone values than the other grilled cuts. While the total value of ketones differed little between the shoulder, lib, and loin cuts (Table 3). Among all ketones, 2-butanone and 2,3-octanedione were the most dominant, which were found in all cuts. The 2,3-butanedione and 6-methyl-5-hepten-2-one were not found in the shoulder, rib, and loin cuts, while 2,3-actanedione was found only in the leg cuts. Generally, ketone flavors can be produced through the oxidation or thermal degradation of fatty acids or amino acids [34]. The flavor notes of ketones are typically desirable, but they may not be significantly important for the aroma contribution of the cooked meat since ketones generally contained high odor threshold values [40]. In this study, ketones were obtained at low concentrations (approximately 8–15% of total peak area). This agreed with the previous study, which reported that salted and ripened goat thigh contained low concentrations of ketones [36]. Thus, it may be assumed that ketones had no greater impact on the overall flavor of grilled goat meat.

Alcohol is the main volatile found in all grilled cuts since it accounted for approximately 20% of the total volatiles. It was found that 1-octen-3-ol existed at a high abundance in all cuts, followed by 1-butanol and 1-octanol (Table 3). In fact, 1-octen-3-ol has a low perception threshold and can confer a light mushroom smell, which is an undesirable odor, to the meat product [41]. Our results were slightly different from other reports that stated that alcohols were obtained at low levels in the cooked goat meat [42]. Alcohols are mainly produced from the oxidative degradation of volatile compounds by lipoxygenase alone or combined with hydroperoxide lyase [34]. However, as compared to other carbonyl compounds, alcohols may not constitute significant contributors to meat flavor because of their high flavor detection thresholds.

There were no differences in the total hydrocarbon compounds found in the grilled rib, loin, breast, and leg goat cuts with % peak areas at 4.61, 3.69, 3.41, and 3.95, respectively, except for the grilled shoulder cuts, which had the highest total hydrocarbons (7.45% peak area). In general, hydrocarbons may not be as important contributors to meat flavor as other carbonyl chemical groups, which can be generated through lipid oxidation or carotenoid degradation [43]. For other lipid-derived compounds, our samples contained four acids and four esters, which accumulated at low concentrations, compared to other groups. Overall, it could be stated that the lipid content and compositions of each cut impacted the various generations of volatiles of the final product. Among all the cuts, the breast cuts contained the highest lipid content as well as UFA, which coincidentally had the highest lipid oxidation products, therefore resulting in the highest accumulation of lipid-derived compounds, particularly aldehydes, ketones, and alcohols.

Additionally, nitrogen- and sulfur-containing compounds were found in all the grilled goat cuts, as shown in Figure 3. These compounds are associated with the Maillard reaction; Maillard-derived volatiles are present in comparatively low amounts in cooked meat when compared to lipid-derived volatiles. Moreover, sulfur volatile compounds can be generated through the reaction of sulfur-containing amino acids (cysteine, cystine, and methionine) with free radicals [44]. All the grilled primal cuts included a high proportion of nitrogen-containing compounds, with % peak areas ranging from 15.01 to 30.44. Regarding the result of the sulfur-containing compounds, the grilled shoulder cuts had the highest overall value of these compounds (10.45% peak area), while the other grilled goat cuts had relatively similar values (4.07–5.61% peak area). The majority of the nitrogen- and sulfur- compounds had a low odor threshold and were thought to be a significant contributor to the desirable meat flavor. This indicates that the shoulder may contribute more flavor to the meat than the other cuts.

## 4. Conclusions

The chemical characteristics (proximate, lipid classes, and fatty acid compositions) of various goat cuts are different, which caused them to produce different lipid oxidation products and volatile compounds. As compared to the other cuts, the breast cuts had the highest fat, diacylglycerol, free fatty acid, and lowest protein contents. Among all the cuts, the highest total UFA was also obtained in the breast cuts. These characteristics led them to be easier to force with lipid degradation/oxidation as indicated by the highest PV and TBARS values. Correspondingly, after grilling, the breast cuts were found to contain the highest lipid-derived volatiles, particularly aldehydes and ketones. Conversely, higher protein-containing cuts such as the shoulder, rib, and loin demonstrated a higher accumulation of nitrogen- and sulfur-containing compounds. Thus, the findings suggest that differences in the chemical properties among the various goat cuts resulted in variations in the lipid oxidation products and volatile compounds, which further resulted in different nutritional values and meat characteristics, particularly flavor. In future studies, other characteristics such as color and texture analyses as well as a sensory evaluation of various cooked primal cuts of goat should be conducted.

## Figures and Tables

**Table 1:** Proximate compositions, cholesterol content, and lipid classes of various goat primal cuts.

Parameters	Shoulder	Rib	Loin	Breast	Leg
Moisture (%)	77.45 ± 0.29 ^b^	78.38 ± 0.30 ^a^	77.76 ± 0.33 ^ab^	76.46 ± 0.34 ^c^	78.39 ± 0.45 ^a^
Protein (%)	19.05 ± 0.52 ^a^	18.30 ± 0.54 ^a^	18.79 ± 0.31 ^a^	16.26 ± 0.40 ^b^	18.26 ± 0.51 ^a^
Fat (%)	2.29 ± 0.31 ^b^	1.90 ± 0.30 ^bc^	1.74 ± 0.21 ^bc^	4.35 ± 0.26 ^a^	1.44 ± 0.25 ^c^
Ash (%)	1.07 ± 0.13	1.08 ± 0.09	1.13 ± 0.05	1.23 ± 0.14	1.01 ± 0.12
Cholesterol (mg/100 g meat)	64.23 ± 3.21 ^ab^	63.65 ± 5.35 ^ab^	62.42 ± 3.19 ^b^	66.88 ± 2.24 ^ab^	69.02 ± 3.30 ^a^
Lipid classes (% of total lipids)					
Triacylglycerol	87.75 ± 2.23 ^a^	86.84 ± 3.04 ^ab^	88.01 ± 2.55 ^a^	82.22 ± 2.92 ^b^	86.22 ± 3.01 ^ab^
Diacylglycerol	1.74 ± 0.15 ^c^	2.03 ± 0.29 ^c^	1.90 ± 0.25 ^c^	4.54 ± 0.22 ^a^	2.95 ± 0.48 ^b^
Monoacylglycerol	ND	ND	ND	0.23 ± 0.09	ND
Phospholipid	9.55 ± 1.03	9.81 ± 0.77	10.01 ± 0.70	9.98 ± 0.69	9.76 ± 1.01
Free fatty acid	0.31 ± 0.06 ^c^	0.50 ± 0.09 ^bc^	0.39 ± 0.08 ^c^	1.92 ± 0.11 ^a^	0.66 ± 0.12 ^b^

Mean ± SD from triplicate determinations. ND = not detected. Different lowercase superscripts in the same row indicate the significant difference (*p* < 0.05).

**Table 2:** Fatty acid profiles of various goat primal cuts.

FA Compositions	Shoulder	Rib	Loin	Breast	Leg
SFA *					
C10:0	ND	ND	ND	0.24 ± 0.03	ND
C12:0	ND	ND	ND	0.66 ± 0.24	ND
C14:0	3.71 ± 0.51 ^a^	1.64 ± 0.44 ^c^	3.17 ± 1.23 ^ab^	1.51 ± 0.50 ^c^	2.30 ± 0.45 ^bc^
C15:0	2.35 ± 0.77	3.41 ± 1.25	2.26 ± 0.30	2.18 ± 0.76	2.55 ± 0.43
C16:0	21.22 ± 1.06 ^a^	24.35 ± 1.54 ^a^	23.55 ± 2.12 ^a^	17.88 ± 1.15 ^b^	18.60 ± 1.23 ^b^
C17:0	8.88 ± 0.96 ^a^	7.00 ± 0.91 ^ab^	6.45 ± 0.84 ^b^	7.66 ± 0.32 ^a^	8.21 ± 0.49 ^a^
C18:0	16.08 ± 1.11 ^a^	14.95 ± 1.25 ^a^	15.62 ± 1.06 ^a^	7.11 ± 1.24 ^b^	5.65 ± 0.78 ^b^
C22:0	0.23 ± 0.04 ^a^	0.09 ± 0.01 ^c^	0.14 ± 0.06 ^c^	1.55 ± 0.10 ^b^	ND
Total SFA	52.47 ± 2.45 ^a^	51.44 ± 4.01 ^a^	51.19 ± 4.53 ^a^	38.79 ± 3.69 ^b^	37.31 ± 2.41 ^b^
MUFA					
C15:1	ND	ND	ND	0.14 ± 0.03	ND
C16:1	ND	ND	ND	0.26 ± 0.10	ND
C16:1n7	0.11 ± 0.08 ^b^	0.29 ± 0.11 ^b^	0.24 ± 0.08 ^b^	2.05 ± 0.08 ^a^	2.22 ± 0.14 ^a^
C17:1	3.66 ± 0.23 ^a^	3.05 ± 0.40 ^a^	3.10 ± 0.33 ^a^	2.03 ± 0.56 ^b^	1.18 ± 0.28 ^c^
C18:1n9	35.55 ± 2.15 ^b^	33.31 ± 2.06 ^b^	31.39 ± 3.22 ^b^	45.45 ± 2.14 ^a^	49.02 ± 4.09 ^a^
C20:1	2.07 ± 0.21 ^b^	2.15 ± 0.33 ^b^	3.06 ± 0.29 ^a^	2.77 ± 0.50 ^a^	3.40 ± 0.43 ^a^
C24:1	1.09 ± 0.08 ^c^	2.53 ± 0.16 ^b^	4.45 ± 0.26 ^a^	1.29 ± 0.23 ^c^	1.03 ± 0.12 ^c^
Total MUFA	42.48 ± 3.05 ^b^	41.33 ± 2.94 ^b^	42.24 ± 4.07 ^b^	53.99 ± 4.14 ^a^	56.85 ± 5.13 ^a^
PUFA					
C18:2n6	2.01 ± 0.22 ^b^	3.50 ± 0.44 ^a^	2.05 ± 0.65 ^ab^	3.02 ± 0.43 ^a^	3.05 ± 0.51 ^a^
C18:3n3	1.46 ± 0.26 ^a^	0.51 ± 0.11 ^b^	1.02 ± 0.29 ^a^	0.24 ± 0.13 ^b^	0.53 ± 0.10 ^b^
C18:3n6	0.20 ± 0.04 ^b^	0.36 ± 0.09 ^a^	0.34 ± 0.09 ^a^	0.09 ± 0.01 ^c^	0.26 ± 0.09 ^ab^
C20:2n6	0.09 ± 0.02	0.10 ± 0.03	0.15 ± 0.06	ND	ND
C20:3n3	0.41 ± 0.05	ND	ND	ND	ND
C20:4n6 (AA) **	ND	ND	0.61 ± 0.04 ^c^	2.04 ± 0.31 ^a^	1.05 ± 0.49 ^b^
C20:5n3 (EPA)	0.24 ± 0.03 ^c^	0.53 ± 0.14 ^b^	1.49 ± 0.25 ^a^	0.67 ± 0.21 ^b^	0.43 ± 0.06 ^b^
C22:6n3 (DHA)	0.11 ± 0.03	0.15 ± 0.05	0.14 ± 0.03	ND	ND
Total PUFA	4.52 ± 0.52	5.15 ± 0.43	5.80 ± 1.04	6.06 ± 0.97	5.32 ± 1.01
Total UFA	47.00 ± 0.04 ^b^	46.48 ± 0.02 ^b^	48.04 ± 0.07 ^b^	60.05 ± 0.04 ^a^	62.17 ± 0.02 ^a^
MUFA/SFA	0.81 ± 0.02 ^b^	0.80 ± 0.02 ^b^	0.82 ± 0.04 ^b^	1.39 ± 0.02 ^a^	1.52 ± 0.03 ^a^
PUFA/SFA	0.08 ± 0.01	0.10 ± 0.03	0.11 ± 0.01	0.15 ± 0.02	0.14 ± 0.01
n-6	2.30 ± 0.02 ^c^	3.96 ± 0.04 ^b^	3.15 ± 0.05 ^b^	5.15 ± 0.07 ^a^	4.36 ± 0.03 ^ab^
n-3	2.22 ± 0.03 ^a^	1.19 ± 0.07 ^b^	2.65 ± 0.06 ^a^	0.91 ± 0.03 ^c^	0.96 ± 0.05 ^c^
n-6/n-3	1.03 ± 0.03 ^d^	3.32 ± 0.04 ^c^	1.19 ± 0.05 ^d^	5.65 ± 0.02 ^a^	4.54 ± 0.03 ^b^

* SFA = saturated fatty acid, UFA = unsaturated fatty acid, MUFA = monounsaturated fatty acid, PUFA = polyunsaturated fatty acid. ** AA = arachidonic acid, EPA = eicosapentaenoic acid, DHA = docosahexaenoic acid. Mean ± SD from triplicate determinations. ND = not detected. Different lowercase superscripts in the same row indicate the significant difference (*p* < 0.05).

**Table 3:** Lipid-derived volatile profiles of grilled goat primal cuts.

Volatile Compounds	Shoulder	Rib	Loin	Breast	Leg
Aldehydes (12)					
3-methyl-butanal	1.52	1.05	2.01	6.02	3.04
2-methyl-2-betenal	ND	1.13	1.55	ND	ND
Acetaldehyde	0.23	0.50	0.93	0.84	0.78
Pentanal	4.02	4.49	4.15	5.06	3.98
Hexanal	10.15	11.09	10.10	12.77	13.08
2-heptanal	1.53	1.06	2.02	1.44	1.78
Octanal	1.40	2.06	1.02	1.15	1.03
2-octenal	ND	ND	ND	0.26	ND
Nonanal	2.18	3.45	2.01	3.44	3.06
2-undecenal	0.22	0.40	0.78	1.56	0.54
4-pentylbenzaldehyde	ND	ND	ND	0.32	0.41
Decanal	1.10	0.78	0.65	1.14	0.96
Total aldehydes	22.35	26.01	25.22	34.00	28.66
Ketones (12)					
Acetone	0.10	0.15	0.09	0.26	0.50
2-propanone	0.65	0.40	0.53	1.51	1.22
2-butanone	4.03	3.45	2.02	2.40	1.88
2,3-butanedione	ND	ND	0.32	2.13	2.16
2-pentanone	1.01	1.41	1.50	0.26	0.50
2-heptanone	1.02	1.16	0.65	0.54	1.22
2,3-actanedione	ND	ND	ND	ND	0.99
6-methyl-5-hepten-2-one	ND	ND	ND	1.01	0.23
2,3-octanedione	2.05	3.14	2.12	5.58	6.04
2-octanone	0.12	0.26	0.10	0.23	0.20
2-nonanone	0.08	0.14	0.50	0.22	0.09
2-decanone	0.06	0.22	0.25	0.09	0.08
Total ketones	9.12	10.33	8.08	14.23	15.11
Alcohols (13)					
Ethanol	0.53	0.26	0.23	0.20	0.35
2-methyl-1-proponol	0.06	0.10	0.14	ND	0.02
1-butanol	3.04	1.55	2.02	3.16	4.07
2-butanol	1.20	2.23	1.06	1.44	1.03
3-methyl-butanol	0.34	0.50	0.45	0.88	0.43
1-pentanol	1.75	1.24	2.03	3.23	2.40
1-penten-3-ol	ND	ND	ND	0.05	0.12
1-hexanol	0.25	0.10	0.42	0.17	0.16
1-heptanol	0.20	0.13	0.14	0.04	0.07
1-octen-3-ol	7.12	11.44	9.28	12.05	12.11
2-ethyl-1-hexanol	1.78	1.23	0.24	1.01	1.44
2-octen-1-ol	0.13	ND	ND	ND	ND
1-octanol	3.72	2.77	2.05	3.06	2.02
Total alcohols	20.12	21.55	18.06	25.29	24.22
Hydrocarbons (10)					
1,1-diethoxy-ethane	0.21	0.05	0.08	ND	ND
Pentane	1.44	1.42	1.30	2.21	2.64
3-methyl-pentane	0.55	0.32	0.27	0.29	0.18
Octane	0.08	0.26	0.19	0.15	0.31
Nonane	0.52	0.89	0.20	ND	0.24
1-nitro-hexane	ND	0.20	0.09	ND	ND
Dodecane	0.34	ND	ND	ND	ND
Tetradecane	1.01	0.08	0.10	0.14	0.13
Toluene	0.55	0.32	0.24	0.17	0.22
Benzene	2.75	1.07	1.22	0.45	0.23
Total hydrocarbons	7.45	4.61	3.69	3.41	3.95
Acids (4)					
Hexanoic acid	1.01	0.53	1.22	0.41	0.98
Octanoic acid	0.17	0.34	0.67	0.07	0.26
Nonanoic acid	0.10	0.09	0.32	0.04	0.26
Tetradecanoic acid	0.25	0.09	0.30	ND	ND
Total acids	1.53	1.05	2.51	0.52	1.50
Esters (4)					
Ethyl acetate	0.15	ND	ND	ND	ND
Hexanoic acid, ethyl ester	0.53	0.16	0.46	1.01	1.43
Decanoic acid, ethyl ester	0.31	0.09	0.21	0.90	0.42
Octadecenoic acid, methyl ester	0.67	0.20	0.34	0.51	0.27
Total esters	1.66	0.45	1.01	2.42	2.12

Expressed as percentage of relative peak area of total peak. ND = not detected.

## Data Availability

Data is contained within the article.
